# Function-driven design of *Bacillus kochii* and *Filobasidium magnum* co-culture to improve quality of flue-cured tobacco

**DOI:** 10.3389/fmicb.2022.1024005

**Published:** 2023-02-16

**Authors:** Xinying Wu, Wen Cai, Pengcheng Zhu, Zheng Peng, Tianfei Zheng, Dongliang Li, Jianghua Li, Guanyu Zhou, Juan Zhang, Guocheng Du

**Affiliations:** ^1^School of Biotechnology, Jiangnan University, Wuxi, China; ^2^Science Center for Future Foods, Jiangnan University, Wuxi, China; ^3^School of Liquor and Food Engineering, Guizhou University, Guiyang, China; ^4^Technical Research Center, China Tobacco Sichuan Industrial Co., Ltd., Chengdu, China; ^5^The Key Laboratory of Carbohydrate Chemistry and Biotechnology, Ministry of Education, Jiangnan University, Wuxi, China

**Keywords:** co-culture, bioaugmentation, *Bacillus kochii*, *Filobasidium magnum*, function-driven design, flue-cured tobacco

## Abstract

Flue-cured tobacco (FCT) is an economical raw material whose quality affects the quality and cost of the derived product. However, the time-consuming and inefficient spontaneous aging is the primary process for improving the FCT quality in the industry. In this study, a function-driven co-culture with functional microorganisms was built in response to the quality-driven need for less irritation and more aroma in FCT. The previous study has found that *Bacillus kochii* SC could degrade starch and protein to reduce tobacco irritation and off-flavors. The *Filobasidium magnum* F7 with high lipoxygenase activity was screened out for degrading higher fatty acid esters and terpenoids to promote the aroma and flavor of FCT. Co-cultivation with strain SC and F7 obtained better quality improvement than mono-culture at an initial inoculation ratio of 1:3 for 2 days, representing a significant breakthrough in efficiency and a reduction in production costs compared to the more than 2 years required for the spontaneous aging process. Through the analysis of microbial diversity, predicted flora functions, enzyme activities and volatile compositions within the mono- and co-cultivation, our study showed the formation of a function-driven co-culture between two strains through functional division of labor and nutritional feeding. Herein, the function-driven co-culture via bioaugmentation will become an increasingly implemented approach for the tobacco industry.

## Introduction

Microbial groups have diverse metabolic capabilities and play an active role in various natural processes. Various functional strains have been screened for application in different industries *via* mono-culture (De Melo Pereira et al., 2020). Recently, co-cultivation has attracted attention because it has the potential to achieve higher biomass, create higher-quality products (Charubin and Papoutsakis, 2019; Shahab et al., 2020), and aid in the biotransformation of harmful substances (Hernández-Adame et al., 2021; Ke et al., 2021), even for multiple demands (Canon et al., 2020). However, the stability of co-cultivation remains the biggest challenge of this methodology. Therefore, developing the appropriate strategies to enhance collaborative relationships between strains is necessary.

Tobacco (*Nicotiana tabacum L.*) is a global economic crop usually made into flue-cured tobacco (FCT). Due to the differences in climate and plant-related soil and agronomic practices in different regions and environments, not all tobacco can be made into high-quality raw materials. Hence, FCT must be stored in an aging warehouse for spontaneous aging under natural conditions. The aging process is essential for improving the quality of FCT in tobacco industry production. During the two-year aging process, the macromolecular components of FCT are degraded or converted into aroma-causing compounds through the synergistic action of microorganisms, enzymes, and chemical oxidation. Nevertheless, some FCT does not still meet product demands after this process due to the low efficiency of spontaneous aging, which becomes the FCT with quality defects to reduce the utilization and increase the cost of raw materials in the enterprise. Consequently, finding an effective method to improve the quality of defective FCT is necessary. Artificial aging using functional strains has recently received increasing attention (Su et al., 2011). In industrial production, the noted quality deficiencies of FCT are primarily irritation, off-flavor, and lack of aroma. Excessive starch in FCT can cause irritation (Han et al., 2010; Wang et al., 2021). The high protein and total nitrogen content are negatively correlated with the off-flavor and irritation of FCT (Cao et al., 2020; Shen et al., 2022). So, the degradation of starch and protein in FCT helps to improve the quality of the final product, in which the formed reducing sugars and amino acids contribute to the aftertaste and sweetness of the product (Feizi et al., 2020). The high fatty acid (HFA) esters (such as linolenic acid, linoleic acid, and palmitic acid esters) could lead to irritation and astringency, and the modest degradation of HFA esters increases the concentration and softness of the smoking gas (Swain and Stedman, 1962; Yang et al., 2018). Terpenoids are important aroma precursors in FCT, as in many plants. Specifically, carotenoids are major terpenoids in FCT that are degraded into various flavors and aromas, such as safranal, beta-damascone, beta-cyclocitral, and megastigmatrienones so on (Leffingwell, 1999).

However, it is usually difficult for a single strain to meet the multiple requirements for quality improvement in FCT simultaneously. Therefore, we established a co-culture to enhance fragrance while reducing irritation using complementary functional strategies. We obtained *Bacillus kochii* strain SC from the tobacco microflora (accession number MZ198211) derived from previous studies (Wu et al., 2021). Strain SC has an excellent ability to secrete neutral protease and alpha-amylase. Although the *B. kochii* SC bioaugmentation enhanced the softness and decreased the irritation of FCT, the aroma still needed to be improved based on the evaluation panel feedback (Wu et al., 2021). Therefore, screening for strains enabling excellent aroma production capacity to compensate for the lack of aroma in FCT is of substantial importance. It has been confirmed that the oxidative degradation of carotenoids in plants involves dioxygenases and that lipoxygenase (LOX, EC 1.13.11.12) plays a significant role in this process (Lyu et al., 2021). LOX is also regarded as a critical enzyme in fatty acid metabolism, one of the main pathways for synthesizing aromas or flavors in fruits (Li et al., 2021). Therefore, it is expected that the screened strains with high LOX incorporated with *B. kochii* SC to produce good quality-enhancing effects.

Because FCT cannot be sterilized with conventional sterilization methods and processed in an open environment, the pure cultivation of functional strains is impossible. So bioaugmentation makes much practical sense in tobacco fermentation. Bioaugmentation aims to inoculate functional microorganisms into the natural biological system in order to enhance the effects of the mixed microbial system (Atasoy and Cetecioglu, 2020). Bioaugmentation has also been applied to improve the quality and yield of traditional foods (Chai et al., 2020; Viesser Jéssica et al., 2020). Herein, we demonstrated a method for the quality improvement of raw materials using bioaugmentation with co-culture.

In the present study, strain F7 with high production of LOX was screened from the tobacco microflora and identified as *Filobasidium magnum*. *F. magnum* is commonly found in the natural environment (Ken et al., 2013; Zhu et al., 2021) and is closely related to fruit flavor formation as the dominant endophyte of fruits (Sayed et al., 2021). *F. magnum* F7 were co-cultured with *B. kochii* SC with the functionally complementary and evaluated with their impact on FCT quality under an optimized mix rate and fermenting time. The potential motivation for improving quality by co-culture was presented by profiling the microbial diversity, functional enzyme activities, and volatile components.

## Materials and methods

### Screening and identification of functional strains

#### Screening

**Figure 1:**
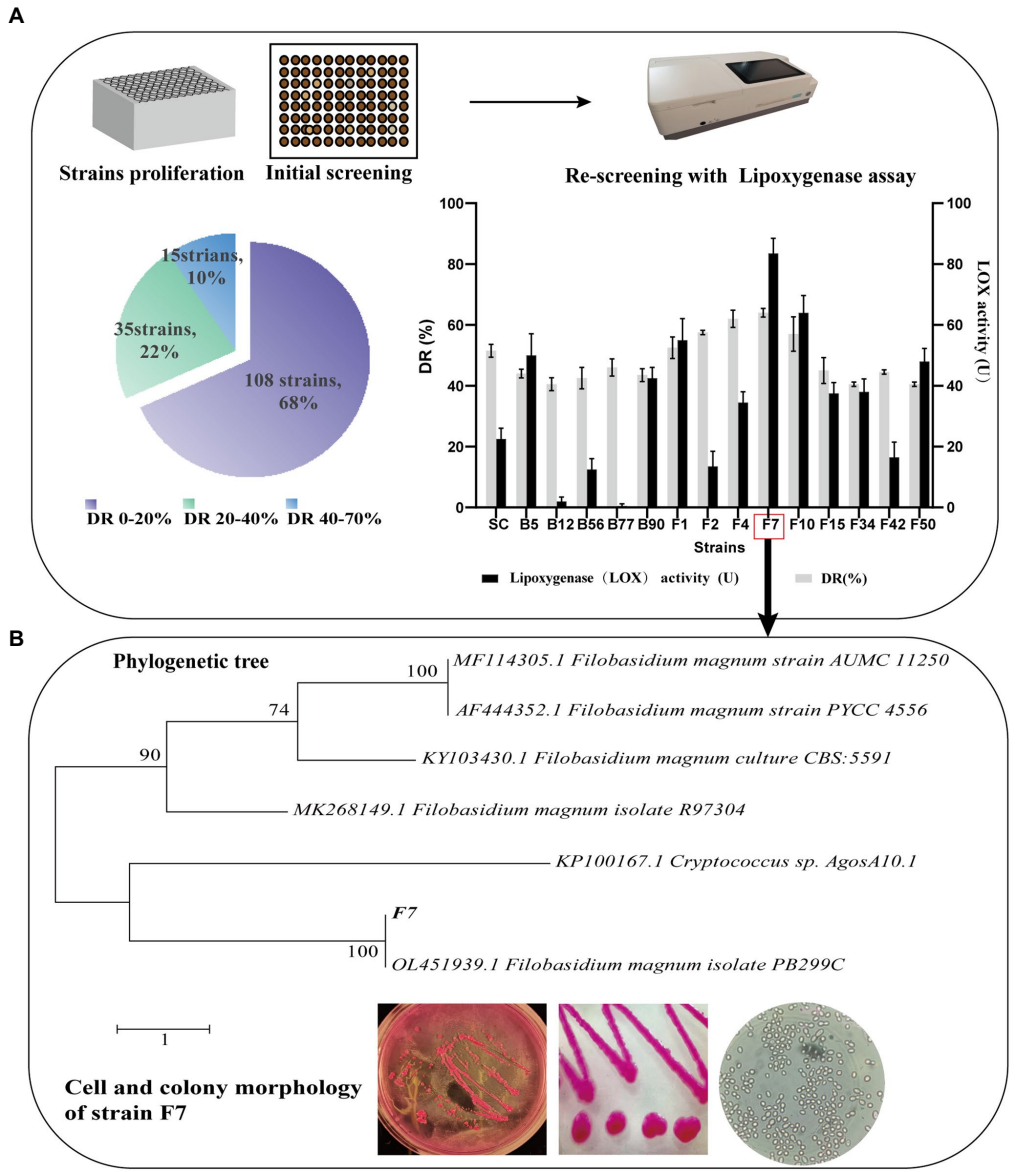
Screening **(A)** and identification **(B)** of functional microorganisms.

The screening protocol for the evaluated functional strains is visualized in Figure 1, including the collection of tobacco microbe mixture, the initial screening of strains, and the re-screening of LOX-secreting strains.

Collection of tobacco microbe mixture: the mixture of tobacco microbes was collected following the previous method (Wu et al., 2021). In brief, a 10 ± 0.1 g of FCT sample was transferred into 200 ml sterilized PBS (phosphate buffer solution; 0.1 mol/l, pH 7.2) and then shocked, sonicated and filtered. The filtrate was centrifuged at 7,000 × g for 10 min. The resulting deposit was resuspended with sterile PBS and then stained with 7-AAD (BD Pharmingen, New Jersey, United States) for 20 min. The multi-strain suspension was filtered through a 40 μm filter and diluted to biomass at an absorbance of 0.3 (optical density: OD600). According to the method described in the previous study (Wu et al., 2021), 1 ml of suspension was transferred into the sample tank of the flow cytometer (FACSARia III cell sorter of BD Biosciences, United States), and single cells were sorted into 96-well plate for culture.

Initial screening of strains: after cultivation, strains formed [colonies in Luria-Bertani (LB) Agar or Bengal Red (BR) Agar]. The strains were then enriched into 96 deep well plates filled with LB broth at 37°C, 400 rpm for 24–72 h, or BR broth at 30°C, 400 rpm for 72–120 h. The addition of FCT extract to the LB and BR media resulted in a yellow–brown color and a strong absorbance of 460 nm measured by spectrophotometry. FCT extract was prepared by reference to Zorn’s method (Zorn et al., 2003). In brief, 20 g of tobacco powder was extracted with a co-solvent of Tween 80 and dichloromethane, and then the solvent was filtered and evaporated off at 40°C and 25 kPa. The residue was re-dissolved in 10 ml of water and sterile-filtered (0.45 μm, Millipore). At last, FCT extract was uniformly dispersed into a sterile medium. After proliferation, the strains with lighter colored medium would be initially screened into a new deep well plate for re-culture regarding their potential to enhance FCT aroma.

Re-screening of LOX-secreting strains: the functional strains were re-screened using a LOX activity assay. LOX activity assay method referred to Lyu’s method (2021). Eighty microliter of crude enzyme supernatant reacted with 920 μl substrate, which was mixed with 10 μl linoleic acid, 5 μl Tween 20, 1 ml NaOH (sodium hydroxide, 0.10 mol/l) and 4 ml PBS (0.20 mol/l, pH 6.5), diluted to 29 ml. The absorbance was kinetically determined at 234 nm. One unit of LOX activity (U) was defined as a change in absorbance of 0.1 per mL of suspension per min. The strain with the highest LOX activity was isolated from the pure culture.

#### Functional strain identification

After morphological and colony identification of the strain with the highest LOX activity, taxonomic identification was carried out. Fungi colony identification was performed using the internal transcribed spacer region (ITS) sequencing. Genomic DNA was extracted with a Fungi Genomic DNA Extraction Kit (Solarbio Science & Technology Co. Ltd. Beijing, China). The following primers were used for ITS1-5.8S rRNA-ITS2 region amplification: ITS1 (5′-TCCGTAGGTGAACC TGCGG-3′) and ITS4 (5′-TCCTCCGCTTATTGATATGC-3′). PCR was performed following Ni’s method (2021) (Rocío et al., 2020), and PCR products were sequenced by Sangon Biotech Co., Ltd. (Shanghai, China). The sequencing data were blasted against NCBI (U.S. National Center for Biotechnology Information) database. The phylogenetic tree of the evaluated strain was mapped in MEGA software (molecular evolutionary genetics analysis, version 7.0.26^1^) using maximum parsimony analysis. The identified functional strain was named strain F7.

### Bioaugmentation of functional strains

In the present study, the FCT with quality defects was used as an object of bioaugmentation and collected from China Tobacco Si Chuan Industrial Co Ltd. (Chengdu County, Sichuan Province, China). This FCT underwent spontaneous aging for 2 years in a factory setting. However, the quality of the FCT still failed to meet production requirements due to high irritation, heavy impurities, and a lack of aroma. The defective FCT evaluated herein was Yunyan 87, among the main varieties of light-flavor FCT widely planted in China. Before bioaugmentation with functional strains, FCT was pre-treated by slicing them into picadura. The bioaugmentation with functional strains was prepared as follows. Single colonies of strain SC or F7 grown on LB/BR agar plates were inoculated into 5 ml broth for further growth. When the growth reached 7–8 log CFU (colony forming units)/mL, the colony’s suspension was mixed thoroughly with FCT at 20% inoculum. Under 85% relative humidity, mono-culture with strain SC and F7 were incubated at 37°C and 30°C, respectively, and co-culture was incubated at 30°C. The culture is stirred once every 4 h. After bioaugmentation, FCT samples were ground into powder in liquid nitrogen and were stored at -80°C for further analysis. Meanwhile, the control group was treated with equal amounts of sterile water. The culture time and proportions of two strains in co-culture were optimized according to the quality evaluation of FCT.

### FCT quality evaluation

We strictly followed the FCT quality evaluation standards delineated within China’s tobacco industry (YC/T138-1998, YC/T496-2014) and national standards (GB/T102212012, GB/T12310-2012). The different tests and descriptive tests are the standards’ primary sensory evaluation methods. Panelists with excellent individual qualifications included two females and five males. The quality evaluations were conducted on a nine-point quality scale. Quality scores lower than four points are considered unacceptable quality, and a score of five to seven is acceptable; when the score is seven or higher, the sample quality matches the superior quality. There were eight evaluation indicators: aromatic intensity, aromatic quantity, pleasant odor, smoke intensity, smoke quantity, softness, sweetness, and aftertaste. The total score of quality evaluation was the sum of all indicators.

### Profiling of microbial diversity

#### DNA extraction and PCR amplification

Referring to Su’s study (2011) with some modifications, 10 ± 0.1 g of sample was added into 200 ml sterilized PBS (0.1 mol/l, pH 7.2) and shaken at 220 r/min, 30°C for 2 h, then sonicated for 5 min and filtered by the sterile absorbent gauze. The filtrate was first centrifuged at 500 × g for 10 min to remove FCT fragments, then repeated the above experimental steps two to three times. The microbial sediment was collected by centrifugation at 10,000 × *g* for 10 min. Then the sediment was resuspended by sterile deionized water. One milliliter of suspension was used to extract the metagenomic DNA from the FCT microflora using the DNeasy PowerSoil Kit.

The metagenomic DNA was used as the template for gene amplification. The V4-V5 region of the bacteria 16S rRNA gene was amplified with barcoded universal primers (515F: 5′-GTGCCAGCMGCCGCGGTAA-3′; 907R: 5′-CCGTCAATTCMT TTRAG TTT-3′). ITS sequencing of the fungal rRNA gene was amplified with the primer (ITS1F: 5′-CTTGGTCATTTAGAGGAAGTAA-3′; ITS2: 5′-GCTGCGTTCTTCATCGATGC-3′). PCR program was set to 98°C for 2 min, 30 cycles of 98°C for 15 s, 55°C for 30 s, 72°C for 30 s, and finally, an extension at 72°C for 5 min. After purification and recovery using magnetic beads, the amplicons were subjected to biological analysis.

#### Bacterial and fungal diversity analysis

Equal amounts of the amplicons were measured with paired-end 2 × 250 bp (base pair) sequencing on the Illlumina MiSeq platform (Illumina, San Diego, CA, United States). Bioinformatic analysis was performed on the raw sequence data2 using QIIME2 software (2019.4^2^; Bokulich et al., 2018). The non-singleton amplicon sequence variant (ASV) taxonomy of the 16S rRNA genes was blasted against the Silva database,^3^ and the ASVs of ITS genes were blasted against the UNITE database.^4^ The sequencing data have been submitted to NCBI (accession numbers: PRJNA762207 for bacterial sequences and PRJNA764574 for fungal sequences). The data randomly extracted from sequences in each sample were flattened to reach a uniform depth for analyzing the relative abundances of ASVs.

#### Microflora function prediction

The PICRUSt2 (Phylogenetic Investigation of Communities by Reconstruction of Unobserved States) software package was used to predict the potential genetic functions based on the 16S rRNA or ITS sequence. The MetaCyc database^5^ was used to predict the primary and secondary metabolic pathways (Dayalan et al., 2019).

### Analysis of starch content

The sample powder was prepared with a grinder (TL-48R, Jingxin, Shanghai, China) at 60 Hz for 90 s. Starch contents in samples were determined using a continuous flow analyzer (Wang et al., 2021). Briefly, 0.2 ± 0.01 g of FCT powder was added to 25 ml of 80% ethanol-sodium chloride saturated solution (at a volume ratio of 3:1) and was ultrasonically treated for 25 min and then centrifuged at 8,000 × *g* for 10 min to remove the pigment. Starch was extracted by adding 15 ml of 40% perchloric acid to the sediment and sonicating it for 15 min. The filtrate was collected after adding another 15 ml of deionized water. The starch in the filtrate was analyzed colorimetrically at 570 nm by reaction with iodine using a Bruker FT-NIR spectrometer (Flyer MATRIX-F, Bruker, Germany) under acidic conditions based on the industry standard (YC/T 216–2013).

### Analysis of enzymatic activity

2.5 ± 0.1 g of sample powder was added into 30 ml sterilized PBS (0.2 mol/l, pH 6.5) and was shocked at 220 r/min, 30°C for 1 h. The supernatant was collected by centrifuging (10,000 × *g*) at 4°C for 10 min. The LOX activity was assayed according to the above method. Neutral protease activity was determined by the Folin color method (Li et al., 2017). One unit of neutral protease activity was defined as 1 μg tyrosine produced from casein per hour at 40°C, pH 7.0. The alpha-amylase activity was determined by DNS (Suraiya et al., 2018). One unit of alpha-amylase activity was defined as 1 mg of reducing sugar produced from soluble starch per minute at 60°C and pH 6.0. Enzyme activity is expressed as activity units per gram of dried sample powder (DW).

### Profiling of volatile compositions

FCT volatiles was profiled using untargeted metabolomics based on HP-SPME-GC-TOF-MS (gas chromatography time-of-flight mass spectrometry with headspace solid-phase micro-extraction). A 2.00 ± 0.01 g FCT powder was placed in the 20 ml headspace bottle with 1 μl internal standard (1 μg/ml tritiated naphthalene dissolved in dichloromethane, Supelco, Aladdin). Volatiles were extracted by SPME fiber assembled with DVB/CAR/PDC (divinylbenzene/carboxyl/polydimethylsiloxane, 50/30 μm) at 60°C for 30 min.

The volatile components of samples were detected with a GC-TOP-MS system (Pegasus BT; LECO, St. Joseph, Michigan, United States) coupled with Agilent 7890A GC and Agilent DB-5MS column (30 m × 250 μm × 0.25 μm, J&W Scientific, Folsom, CA, United States). The chromatographic conditions c the column flow was 1 ml/min; the inlet temperature was 250°C; the heating temperature was maintained at 40°C for 2 min and then increased to 250°C at a rate of 10°C/min for 6 min; the ion source adopted the electron bombardment model, the electron energy was 70 eV; the transmission line and ion source temperature were 280°C and 210°C, respectively; the mass spectrometry data were retrieved in full-scan mode (range, 33–400 atomic mass units at a rate of 3 specs/s), and the data acquisition rate was 10 specs/s.

The raw peaks extraction, data baseline filtering, and calibration, alignment, deconvolution analysis, peak identification, and integration were executed with the support of Chroma TOF 4.3X software (LECO Corp., St. Joseph, MI, United States) and the LECO-Fiehn Rtx5 database (Li et al., 2020). The compositions matched NIST (U.S. National Institute of Standards and Technology) and Wiley library^6^) databases regarding retention indices and mass spectra. Identified compounds with matching scores of over 700 were further analyzed. Peaks detected in <50.0% of samples or RSDs (relative standard deviations) of peak areas >30.0% were removed. Using MetaboAnalyst5.0 software,^7^ a PLS-DA (partial least squares-discriminant analysis) model of samples was constructed to identify differential compounds according to VIP (Variable importance of projection) values of >1 and *p*-values of <0.05.

### Statistical analysis

Data were displayed as mean ± standard deviation. The volatile components were analyzed using six replicate samples, and other results were performed at three parallels. Bar charts and sample variances were depicted using GraphPad Prism 6 software (GraphPad, San Diego, CA, United States).

## Results

### Construction of co-cultivation

With the goal of co-cultivation with *B. kochii* SC to comprehensively improve the quality of FCT, we screened functional microorganisms with high LOX activity from the FCT microflora. Following the screening protocol (Figure 1), 158 well-grown strains were obtained from colonies grown in 96 shallow-well plates. In the initial screening, the decolorization of the medium by the strains was compared with uninoculated samples. The percentage of color reduction was defined as the decolorization rate (DR). Fifteen strains achieved a high DR of 40–70%. Strain F7 had the highest DR and LOX activities of 64 ± 5.6% and 83.5 ± 2.2 U/ml, respectively. As a result, strain F7 was selected as a study subject.

The phylogenetic tree of strain F7 and microscopic imaging under the magnification of 100 × 1.25 are shown in Figure 1. The cells of strain F7 were spherical or ovoid, and some cells showed asexual reproduction with monopolar budding, which was the distinctive feature judged as yeast. The colonies of strain F7 were characterized by smooth, convex, rounded edges, moist, sticky, and easy to pick up. Strain F7 colonies were milky white when grown on malt extract agar. When strain F7 was cultured in RB, the colonies appeared purplish red, due to the entry of medium pigments into the cells. The phylogenetic tree was constructed by the neighbor-joining method with the bootstrap values at the edge of each node. The blast analysis of ITS1 gene domain sequences of strain F7 reveals a closer related *F. magnum*. Therefore, strain F7 is identified as a strain of *F. magnum*, and the sequence has been submitted to the GenBank database (NCBI; accession number, MZ351194).

### Effects of bioaugmentation on quality attributes

**Figure 2:**
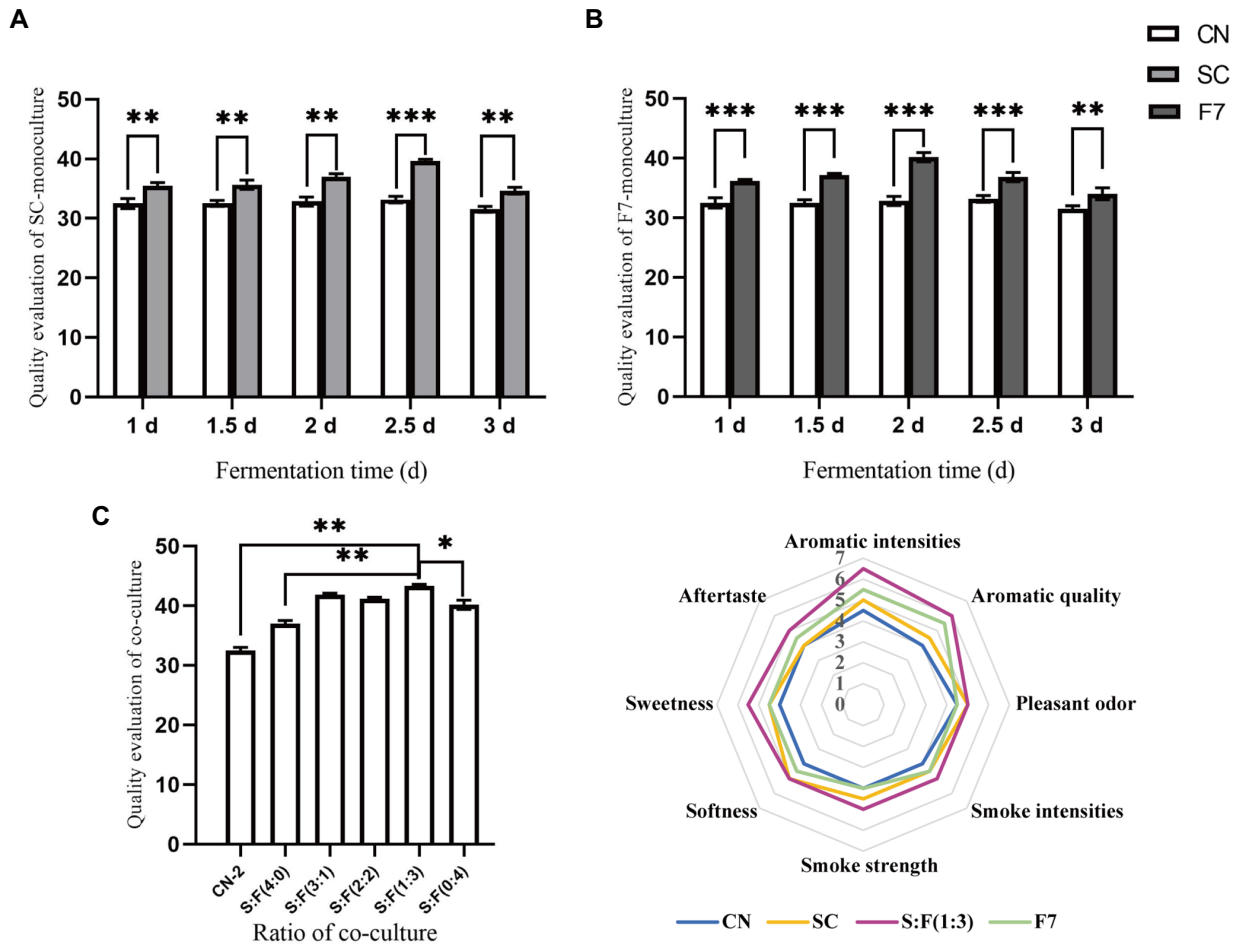
Dynamic analysis of FCT quality response to *Bacillus kochii* SC mono-culture **(A)**, *Filobasidium magnum* F7 mono-culture **(B)**, co-cultivation at different mixing ratios **(C)**, and sub-quality indicators evaluation of co-cultivation **(D)**. CN represents the control group. SC represents *B. kochii* SC mono-culture samples. F7 represents *F. magnum* F7 mono-culture samples. S:F represents the sample of *B. kochii* SC-*F. magnum* F7 co-culture. Error bars represent the standard deviation of the samples (triplicate). *p* value represents by an asterisk “*” (*: 0.01 < *p* ≤ 0.05; **: 0.001 < *p* ≤ 0.01, ***: *p* ≤ 0.001; ns: no significant difference).

We dynamically studied the changes in quality attributes of mono and co-cultivation with different fermentation times. Following the quality evaluation criteria of FCT, difference and descriptive tests with negative controls were performed in our study to highlight the quality-enhancing effect of bioaugmentation with functional strains on FCT with quality defects. Mono-culture SC and F7 fermentation for 2.5 days and 2 days, respectively, obtained the highest quality evaluation scores, as shown in Figures 2A,B, with statistically significant differences between the bioaugmentation and control samples (*p* < 0.001, two-tailed). The quality evaluation score was the highest when SC and F7 were co-cultivated for 2 days at the initial inoculation ratio of 1:3 (Figure 2C). There was also a statistically significant quality enhancement in co-culture compared to the control and mono-culture samples (*p* < 0.001, two-tailed). According to Figure 2D, bioaugmentation with SC favored softness and pleasant odor, while mono-culture of F7 contributed to aroma intensity and aroma quality. Based on functional complementarity, co-cultivation obtained comprehensive quality improvements, obviously enhancing aroma-related attributes, softness and pleasant odor, simultaneously increasing the sweetness and aftertaste of the FCT. The control group had a score of <5 for each evaluation attribute, while the test group using co-culture scored over 5. The aroma intensity and aroma quality increased significantly, reaching over 6 points.

### Motivation related to quality improvement

The intervention of functional strains altered the diversity of natural consortia on tobacco and the activity of secreted enzymes and additionally affected the chemical composition of FCT through metabolism and/or catalysis.

#### Changes in microbial diversity

**Figure 3:**
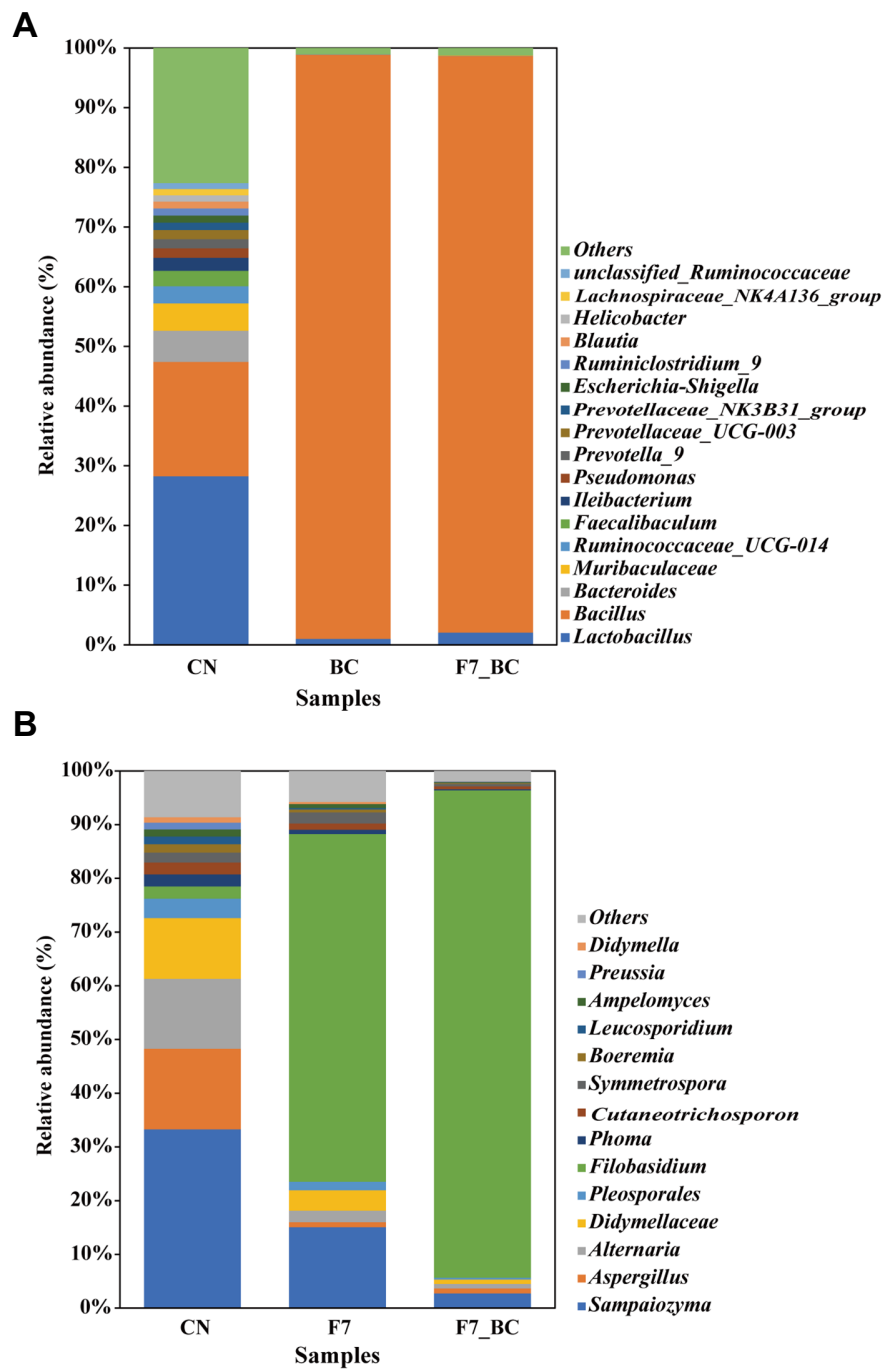
Analysis of bacterial **(A)** and fungal **(B)** diversity.

This study applied microbial assembly from different phylogenetic groups to bioaugmentation. A total of 413,098 16S rRNA and 384,294 ITS1-5.8S rRNA-ITS2 high-quality sequences were obtained individually from 12 samples. Each deduplicated sequence was defined as ASVs. After data was flattened using a rarefaction method, the relative abundance of ASVs was observed at the same sequencing depth (18,232 bacterial and 5,443 fungal ASVs per sample) (Supplementary Tables 1, 2). The microbial diversity of the controls differed statistically significantly from that observed within bio-inoculation samples (Figures 3A,B). Based on the alpha-diversity analysis of bacterial and fungal communities (Supplementary Tables 3, 4), Goods-coverage indices are higher than 99%, which suggests the sequencing results with high coverage in a gene library of samples. Observed species and Chao1 indices can characterize data richness, whereas Shannon and Simpson indices indicate the diversity and evenness of a sample (Su et al., 2020). Consequently, the bio-inoculation samples had less complex diversity and lower richness than the control group (*p* < 0.05) due to the intervention of functional microorganisms.

Regarding the relative abundance of functional strains, there was no statistically significant difference in bacterial flora between the bio-inoculated samples. In contrast, there was a significant difference in fungal flora between mono-culture with strain F7 and co-culture with strain F7 and SC, which should be due to the intervention of strain SC in the co-culture. According to the findings presented in Figures 3A,B, 18 bacterial genera and 14 fungal genera had a relative abundance greater than 1% in the control group spontaneously fermented at 85% relative humidity and 30°C for 2 days. However, there was less complex diversity in bio-inoculation samples. The relative abundance of *B. kochii* SC was close to 100% because of its outstanding growth properties. The relative abundance of strain F7 was elevated from 2% (control group) to 90.73% (co-cultivation). Strain F7 developed more readily in co-culture (90.73%) than in monoculture (64.68%), suggesting that strain SC was a good promoter of strain F7. Consequently, additional research is needed to verify how strain SC promoted the growth of strain F7. In bioaugmentation, functional strains dominate, contributing to serving the purpose of bioaugmentation (Viesser Jéssica et al., 2020).

**Figure 4:**
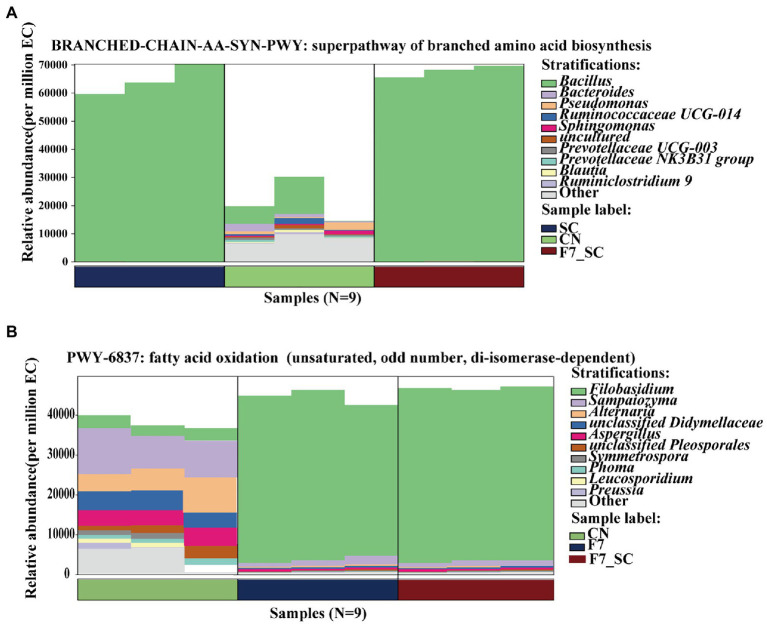
Maps of the genera composition in the differential MetaCyc metabolic pathway of bacterial **(A)** and fungal **(B)** consortia. CN represents the control group. SC represents *B. kochii* SC mono-culture. F7 represents *F. magnum* F7 mono-culture. F7-SC represents *F. magnum* F7-*B. kochii* SC co-culture.

PICRUSt2 software was used to predict the functional abundance of consortia according to the marker gene sequences. According to Caspi R’s study (2008), the prediction of MetaCyc metabolic pathways approached the reliability of macroeconomic analysis (Caspi et al., 2008). Biosynthesis and degradation/ utilization/ assimilation metabolism pathways had a higher relative abundance than the others as shown in Supplementary Figure 1. Pathways associated with microbial growth, including carbohydrate degradation, glycolysis, and amino acid synthesis, have a fairly high relative abundance in bacterial communities. The pathways associated with flavor formation, including secondary metabolite degradation in bacterial flora and fatty acid/lipid degradation in fungal flora, showed a higher abundance. Analyzing important differential metabolic pathways with genera composition in Figures 4A,B, *Bacillus* showed a high abundance of amino acid synthesis pathways, consistent with its high protease activity phenotype, and *Filobasidium* could promote the oxidative pathway by secreting a highly active LOX. Unsaturated, saturated C6 and C9 volatile aldehydes/alcohols are generated through the LOX pathway, essential flavor contributors to fruits, vegetables, and herbs (Wilfried et al., 2008).

#### Dynamic changes in functional enzyme activity

**Figure 5:**
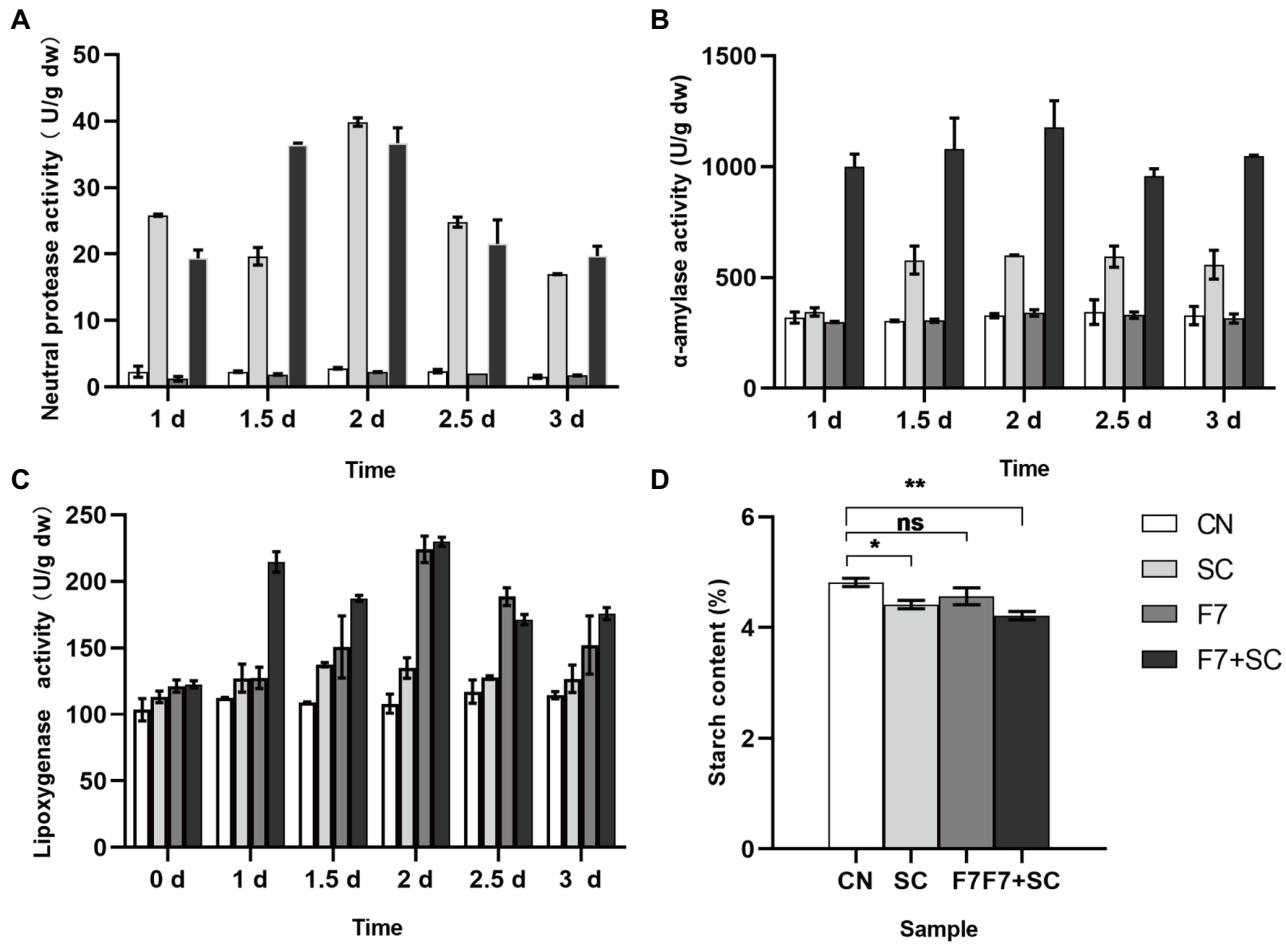
Dynamic analysis of neutral protease activities **(A)**, alpha-amylase activities **(B)**, lipoxygenase (LOX) activities **(C)**, and starch content **(D)** response to bio-inoculation. CN represents the control group. SC represents *B. kochii* SC mono-culture. F7 represents *F. magnum* F7 mono-culture. F7-SC represents *F. magnum* F7-*B. kochii* SC co-culture. Error bars represent the standard deviation of the samples (triplicate). *p* value represents by an asterisk “*” (*: 0.01 < *p* ≤ 0.05; **: 0.001 < *p* ≤ 0.01, paired, *t*-test; ns: no significant difference).

As shown in Figure 5, the enzymatic activity of neutral protease, alpha-amylase, LOX behaved differently in mono-culture and co-cultivation. All samples showed the highest activities of enzymes when fermented for 2 days, with low enzyme activities found in the control group. Our study found high neutral protease and alpha-amylase activity in strain SC mono-culture and co-culture. However, there was no statistical difference between the control and the mono-culture with strain F7 (Figures 5A,B). It could be assumed that strain F7 did not secrete neutral protease and α-amylase. Collaboration of microbial communities may lead to higher α-amylase activity in co-culture than in mono-culture, but additional research is needed. LOX activity could be detected in all samples, but the activity in the mono-, co-culture with strain F7 was higher than in other samples (Figure 5C). Strain F7 was able to secrete LOX, consistent with the predicted results of colony function (Figure 4B). In Figure 5D, starch degradation was higher in the mono-, co-culture with strain SC, while there was no statistically significant change in the mono-culture with strain F7. The overall high enzyme activity in co-cultivation should result from the mutual promotion between the two strains, which may be why co-cultivation can result in substantial quality improvement.

#### Changes in flavor-associated volatile composition

**Figure 6:**
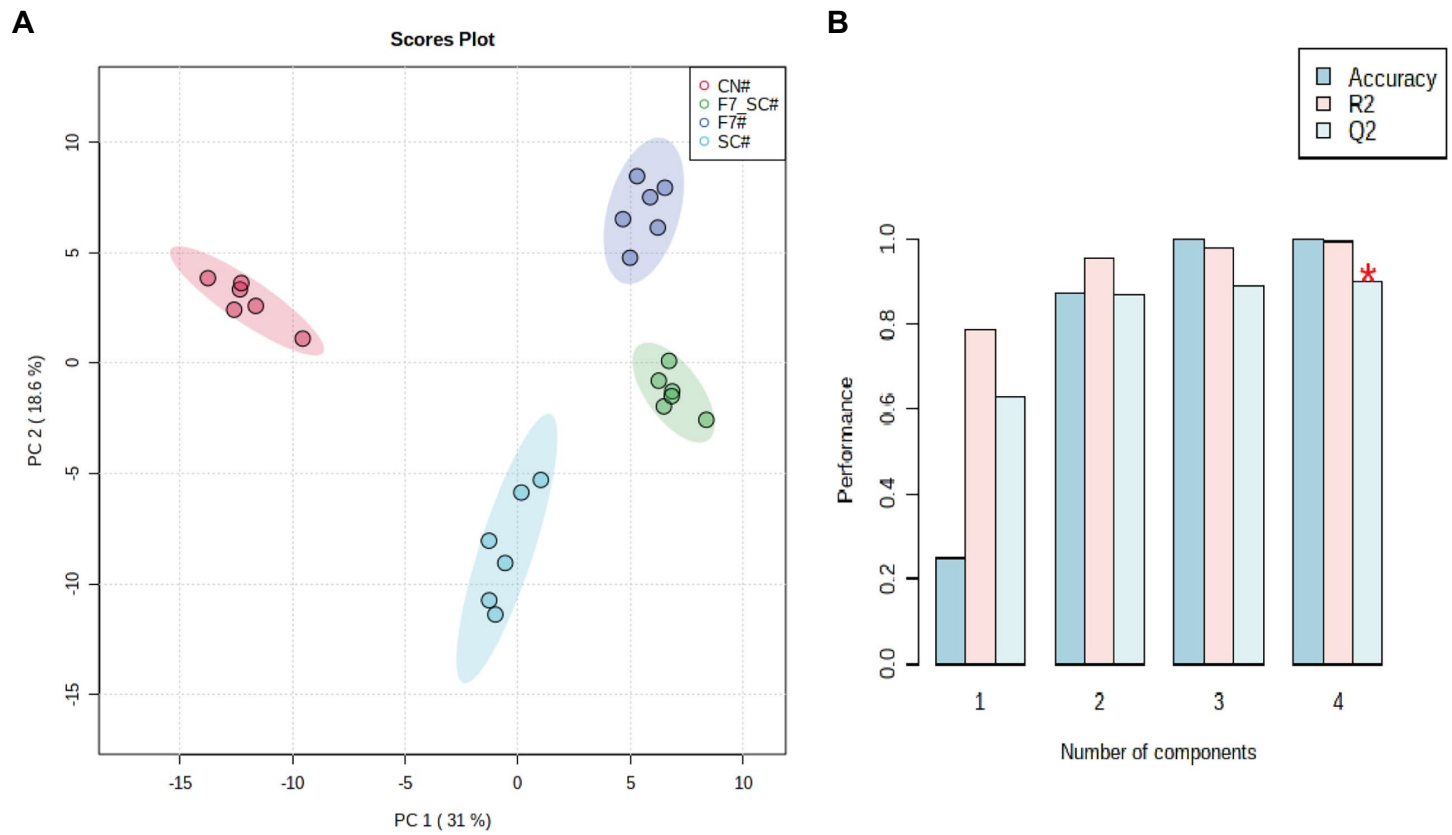
Profiling the volatile compositions in FCT with bio-cultivation. Principal component analysis **(A)** and cross-validation of PLS-DA (R2:0.9945; Q2:0.9355) of the volatile compositions in FCT with bio-cultivation **(B)**.

The flavor-associated volatile compositions have an essential impact on the quality of FCT (Yin et al., 2016). As the main conclusion of our manuscript titled “Profiling the role of microorganisms in quality improvement of the aged flue-cured tobacco” (Submitted for publication), HFA/lipid metabolism, terpenoid degradation, and the formation of Maillard reaction products are the key metabolic pathways involved in the quality improvement of FCT during spontaneous aging. 191 volatiles volatiles were detected using the untargeted metabolomes based on HP-SPME-GC/MS. There were 54 carbonyl compounds, 37 alkanes and alkenes, 35 heterocyclic compounds, 28 esters, 15 aromatic hydrocarbons, 15 alcohols and phenols and 10 acids (Supplementary Table 5). Samples can be distinguished within 95% confidence intervals using unsupervised PCA (principal components analysis) (Figure 6A). In the present evaluation, two principal components contribute 50.9% of the total variance. There is an excellent distinction between the control and bioaugmented groups. In addition, we constructed supervised PLS-DA models. Cross-validation showed that these models had good predictive without over-fitting (R2Y and Q2 were close to 1, as shown in Figure 6B). Given the criteria of VIP values of >1.0 and *p*-values of <0.05, 64 differential metabolites were identified, including 17 compounds related to terpene metabolism, 13 compounds related to fatty acid and lipid metabolism and 13 compounds related to Maillard reaction (Supplementary Table 6).

**Figure 7:**
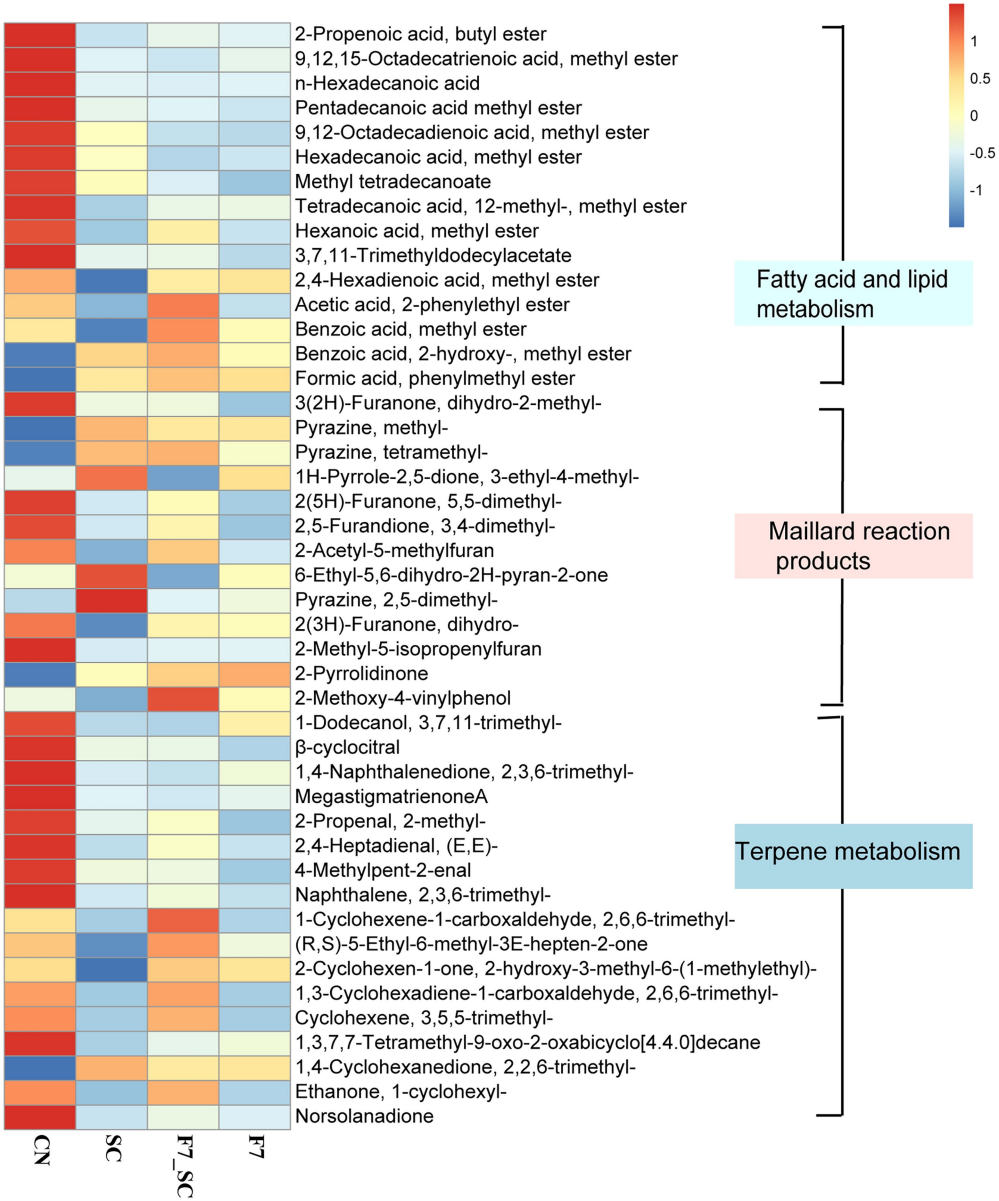
Heat map of key differential metabolites between control and bio-cultivation groups. CN represents the control group. SC represents *B. kochii* SC mono-culture. F7 represents *F. magnum* F7 mono-culture. F7-SC represents *F. magnum* F7-*B. kochii* SC co-culture.

The heat maps of some differential metabolites are shown in Figure 7. In fatty acid and lipid metabolism, *Filobasidium* showed a high capacity for fatty acid oxidation that promoted the degradation of HFA esters. HFA esters include pentadecanoic acid methyl ester, hexadecanoic acid methyl ester and 9,12,15-octadecatrienoic acid methyl ester and so on. Meanwhile, simple esters have the highest content in the bioaugmentation with strain F7_SC and F7, in which 2-phenylethyl acetate had the highest content in co-culture; Most products related to Maillard reaction were at their highest level in bioaugmentation with SC. Terpenoid metabolism products are essential flavors in tobacco, especially the degradation products of carotenoids and cembrene (Ni et al., 2021). However, strain SC and strain F7 both showed the capacity to promote the degradation of aromatic precursors and the accumulation of aromas in terpenoid metabolism. While F7 demonstrated a better capacity than SC in the present study.

## Discussions

In our study, flow cytometry is a powerful method for screening and isolating single cells through high-throughput equipment compared to traditional plate isolation methods (Vitelli et al., 2021). *Via* flow cytometry, the obtained library of tobacco microbes can provide a screening source for functional microbial. The purpose of the initial screening was to obtain microorganisms that could lighten the color of the medium added FCT extract. Pigment in the FCT extract consists of polyphenols, carotenoids, and rutinosides. These compounds are essential precursors of flavor and aroma (Maldonado-Robledo et al., 2003) and transformed or degraded by the action of enzymes or microorganisms. Which leads to a color change in the medium. Thus, the initially screened strains were considered to have the potential to enhance FCT aroma. Based on the ability of the strain to secrete LOX and the effect on FCT flavor, *F. magnum* strain F7 was re-screened. *F. magnum* is one of the dominant genera of fungi in microbial communities of FCT through diversity analysis described in our previous study (Wu et al., 2021). *F. magnum* was also found in the Lhalu Wetland and the rhizosphere soil of the Hami melon orchards in Xinjiang, China (Guo et al., 2018; Zhu et al., 2021). *F. magnum* has also been isolated from the larval midgut of stag beetle, which could degrade biodegradable plastic films (Ken et al., 2013). It was likewise found as an endophytic fungus in the vineyard environment and was closely associated with the formation of grape flavor (Sayed et al., 2021). Unfortunately, we could not conclude whether strain F7 was a tobacco endophyte. With our limited knowledge, a lipoxygenase-producing *Filobasidium* was first screened from FCT *in situ* to promote aroma.

The evaluated test object was FCT with quality defects that had not yet met quality demands. The highest quality score was obtained in bioaugmentation with strain SC and F7 co-cultured for 2 days at the initial inoculation ratio of 1:3. Consequently, the FCT with quality defects (control group) was upgraded from unacceptable quality to acceptable quality to become the qualified raw materials (test group). It was a noticeable and significant improvement in quality for the evaluated FCT. The apparent effect of co-culture on FCT quality implied a synergistic relationship between the two strains and the native consortia. Similarly, Chai et al. (2020) enhanced acetoin accumulation in vinegar fermentation through bioaugmentation of *Lactobacillus casei* and *Acetobacter pasteurianus*, with an initial ratio of 1:1. Canon et al. (2020) developed co-cultures of functionally complementary lactic acid bacteria to ferment a new food with target flavor attributes. It shows that assembling different functional strains through co-culture can achieve better results than those obtained through mono-culture. Good quality improvement was achieved in only 2 days with the co-culture bioaugmentation method. This represents a significant breakthrough in efficiency and a reduction in production costs compared to the more than 2 years required for the spontaneous aging process.

To understand the motivation of co-culture in promoting quality, we compared the differences between the control and bioaugmentation group (mono- and co-cultivation) from different dimensions. Microbial diversity analysis indicated that the intervention of functional strains decreased the diversity and richness of microbial community. Strains F7 and SC dominate overwhelmingly in the flora. Meanwhile, the relative abundance of strain F7 in the co-culture was higher than in the mono-culture. *Bacillus* is ubiquitous and can produce diverse enzymes to degrade different organisms, such as alpha-protease (Yogesh and Halami, 2015), amylase (Ullah et al., 2021) and lipase (Adetunji and Olaniran, 2021). *F. magnum* could secrete lipase and pectinase (Li et al., 2017). In the present study, the high activities of neutral protease and α-amylase in strain SC promoted the degradation of protein and starch into small molecular carbohydrates and amino acids, which provided more nutrients for the growth and metabolism of F7 in the co-culture.

Meanwhile, the analysis of flora genes functions, enzyme activities, and various components in the mono- and co-culture modes suggested the excellent performance of the co-culture based on function-driven design. Changes in the composition resulted from microbial metabolism and created a change in quality characteristics. As shown in Figure 2, the bioaugmentation with SC enhanced the attributes of softness and pleasant odor, while F7 mono-culture contributed to the intensities and quality of aroma. Co-cultivation with two functional strains obtained comprehensive quality improvements. *F. magnum* F7 with high LOX might affect fatty acid, lipid metabolism and terpene metabolism. *B. kochii* SC with high proteases and amylases mainly promoted the accumulation of Maillard reaction products by accelerating the formation of amino acids and reducing sugars. The co-cultivation with two strains achieved a higher accumulation of flavor-related substances by complementary functions.

In Figure 7, HFA esters can degrade into volatile carbonyl compounds (Han et al., 2010) to alleviate irritation and off-flavor, and promotes the accumulation of flavor-related degradation products. 2-phenylethyl acetate is a very valuable flavor compound that provides a stronger fruity character in wine (Viana et al., 2009). Benzoic acid methyl ester is a food-grade oral favor additive (Zheng et al., 2021). Most Maillard reaction products can offer a sweet, nutty, or popcorn-like flavor (Hinneh et al., 2018), in which 1H-Pyrrole-2,5-dione, 3-ethyl-4-methyl- has been reported as a flavor marker in dark tea (strongly associated with an aged fragrance) and 2,5-dimethylpyrazine offers a cocoa flavor (Zhang et al., 2021). As well, the degradation of terpenoids contributes to the lessening of irritation and the formation of flavors. Cembrene could be degraded into solanone with a peculiar flavor and mellowed aroma (Zorn et al. 2003; Popova et al., 2020). 2,6,6-trimethyl-1,3-cyclohexadiene-1-carboxaldehyde (also known as safranal) increases the sweetness and has been identified as an aroma-active compound in dark tea (Zhang et al., 2021). 2,6,6-trimethyl-1-cyclohexene-1-carboxaldehyde and beta-damascone are important degradation products of carotenoids and have floral and fruity scents (Popova et al., 2020).

Through the analysis of microbial diversity, gene function, enzyme activity and volatile compositions within the mono- and co-cultivation, our study showed the formation of a good co-culture between two strains through functional division of labor and nutritional feeding. It took only 2 days of bioaugmentation with co-cultivation to obtain comprehensive quality improvements in the study subject, clearly enhancing aroma-related attributes, softness and pleasant odor, simultaneously increasing the sweetness and aftertaste of FCT. However, functional strains were overwhelmingly dominant in the flora through the bioaugmentation technique. The impact of other microorganisms on the composition of FCT needs to be studied more. We demonstrated that a co-culture method could more effectively meet target needs than previous approaches using a function-driven design. Bioaugmentation with co-cultivation will become a promising approach to the economically targeted promotion of the quality of raw materials suitable for industrialized production with rough handling.

## Data availability statement

The datasets presented in this study can be found in online repositories. The names of the repository/repositories and accession number(s) can be found in the article/Supplementary material.

## Author contributions

XW: data curation, methodology, formal analysis, and writing — original draft. WC: methodology and data curation. PZ: methodology and investigation. ZP: writing-editing and data visualization. TZ: data visualization. DL: resources and funding acquisition. JL: writing — conceptualization and reviewing. GZ: data visualization. JZ: writing — editing and supervision. GD: funding acquisition and writing — reviewing. All authors contributed to the article and approved the submitted version.

## Funding

Our work was funded by the 2020 Major Science and Technology Special Project of China Tobacco Corporation (110,202,001,040 XJ-02) and the Major Science and Technology Special Project of China Tobacco Sichuan Industrial Co., Ltd. (KJSB201808020001).

## Conflict of interest

WC, PZ, and DL were employed by China Tobacco Sichuan Industrial Co., Ltd.

The remaining authors declare that the research was conducted in the absence of any commercial or financial relationships that could be construed as a potential conflict of interest.

## Publisher’s note

All claims expressed in this article are solely those of the authors and do not necessarily represent those of their affiliated organizations, or those of the publisher, the editors and the reviewers. Any product that may be evaluated in this article, or claim that may be made by its manufacturer, is not guaranteed or endorsed by the publisher.

## Footnotes

^1^ https://www.megasoftware.net

^2^ https://qiime2.org

^3^ https://www.arb-silva.de/

^4^ https://unite.ut.ee/

^5^ https://metacyc.org/

^6^ https://onlinelibrary.wiley.com

^7^ https://metaboanalyst.ca
