# Supplementary figures and images for: Function-driven design of Bacillus kochii and Filobasidium magnum co-culture to improve quality of flue-cured tobacco
Source: Front Microbiol. 2023 Feb 16;13:1024005. doi: 10.3389/fmicb.2022.1024005 (PMC9978371; doi:10.3389/fmicb.2022.1024005)

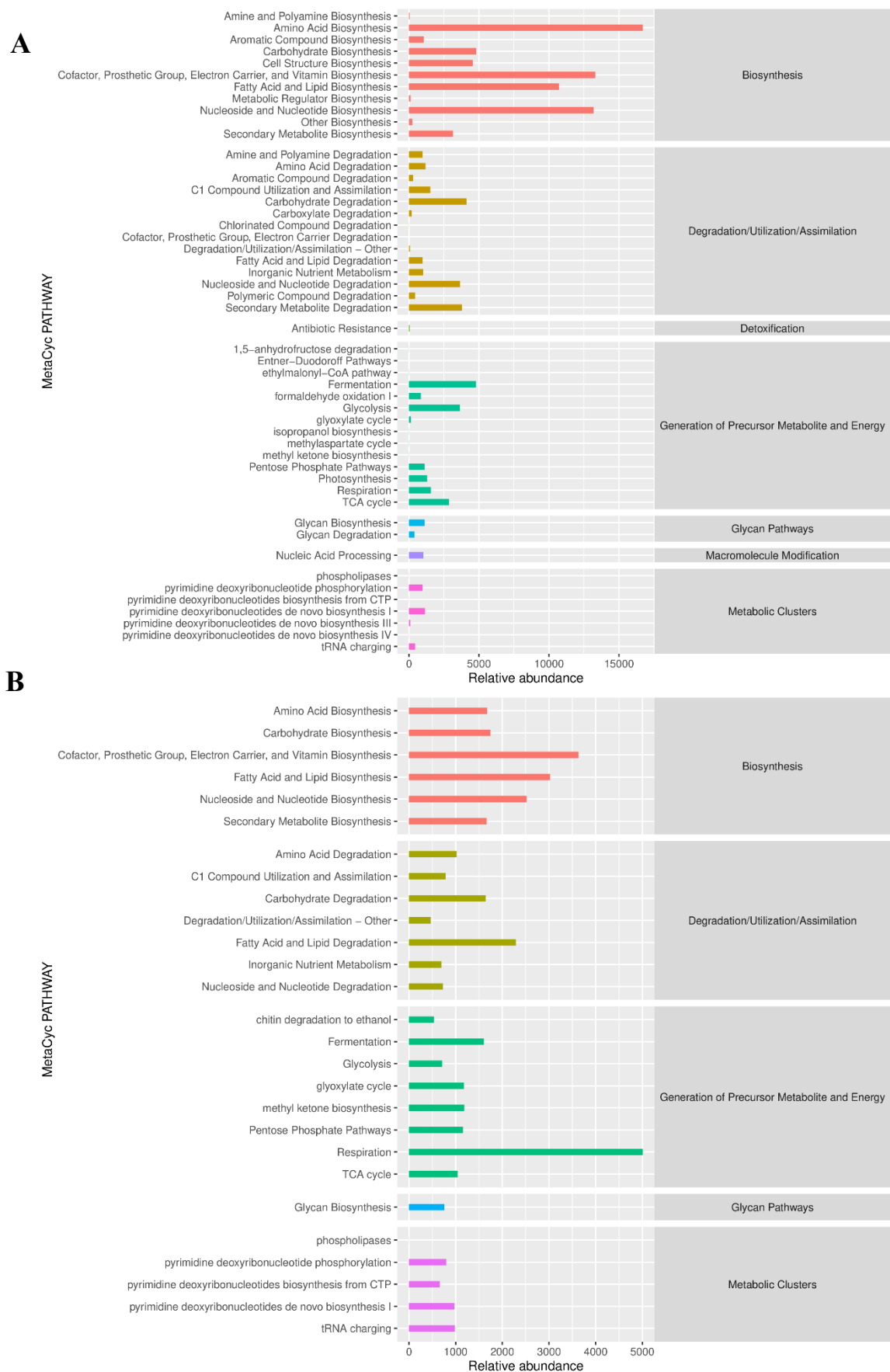

Supplementary Figure 1. Prediction of bacterial (A) and fungal (B) flora function.

Supplement: Supplementary file 1 [file Data_Sheet_1.PDF]
